# Supplementary material for: She1 affects dynein through direct interactions with the microtubule and the dynein microtubule-binding domain
Source: Nat Commun. 2017 Dec 15;8:2151. doi: 10.1038/s41467-017-02004-2 (PMC5732302; doi:10.1038/s41467-017-02004-2)
Supplement: Supplementary file 1 — Supplementary Information [file 41467_2017_2004_MOESM1_ESM.pdf]

SUPPLEMENTARY FIGURES AND FIGURE LEGENDS

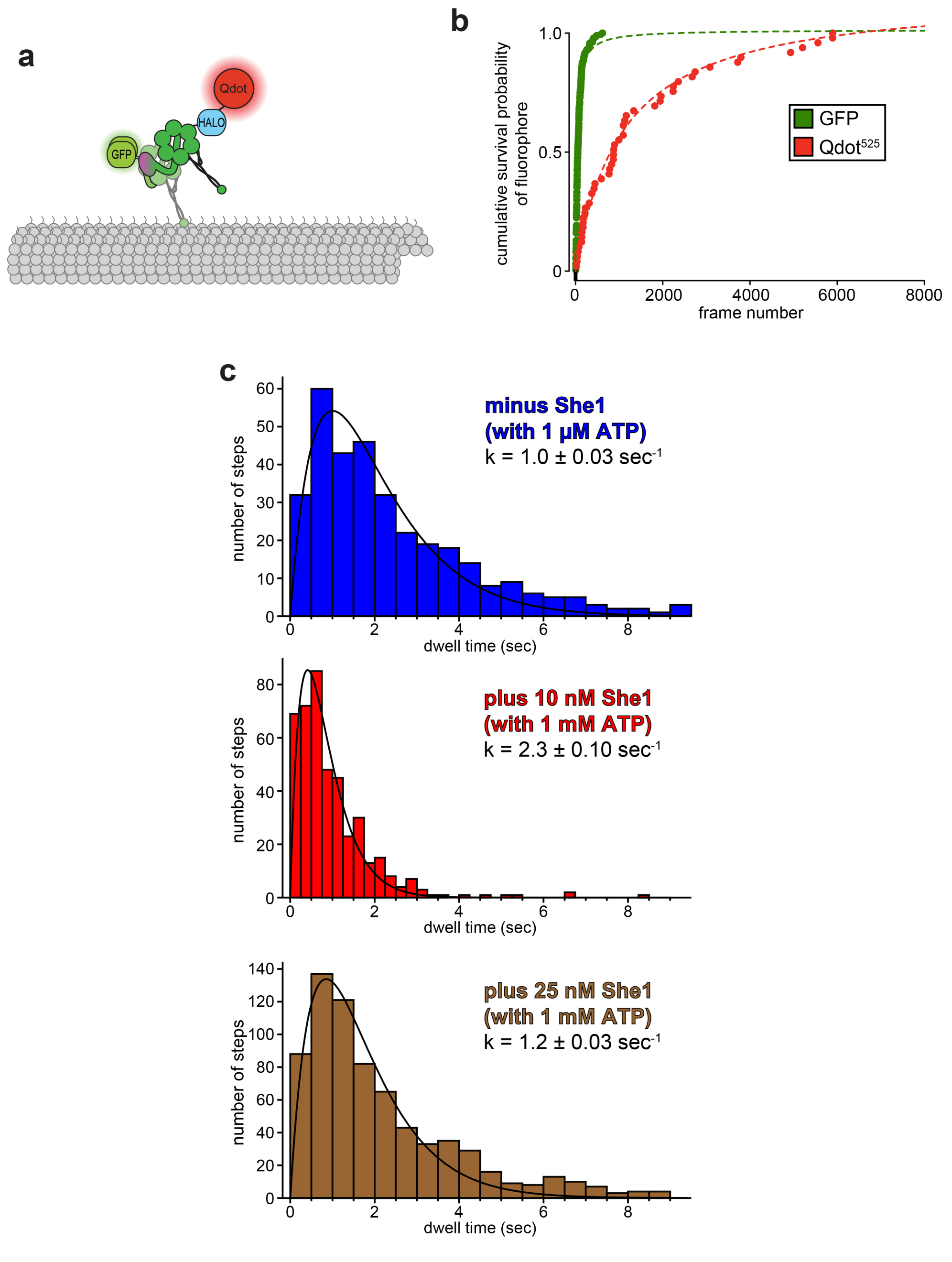

Supplementary Figure 1. **She1 increases dwell time between individual steps of dynein.** (a) Cartoon representation of the GFP-GST-dynein$_{331}$-HALO-Qdot^{525} used in the stepping assays. (b) Plots and fits of the cumulative survival probabilities of the N-terminal GFP and the C-terminal (motor) HALO tag-coupled Qdot^{525}. Note the large difference in photostability between the two fluorophores (see Methods; n ≥ 190 motors for each). (c) Histograms of dwell times (duration

between individual steps) for motor domain labeled (via $Qdot^{525}$) GST-$dynein_{331}$ in the absence or presence of She1, and with either 1 μM or 1 mM ATP, as indicated. The histograms were fit to a convolution of two exponential functions [$tk^2exp(-kt)$] with equal decay constants, k, which reflects the number of steps taken per second[1,2] (k ± standard error of the fit is shown).

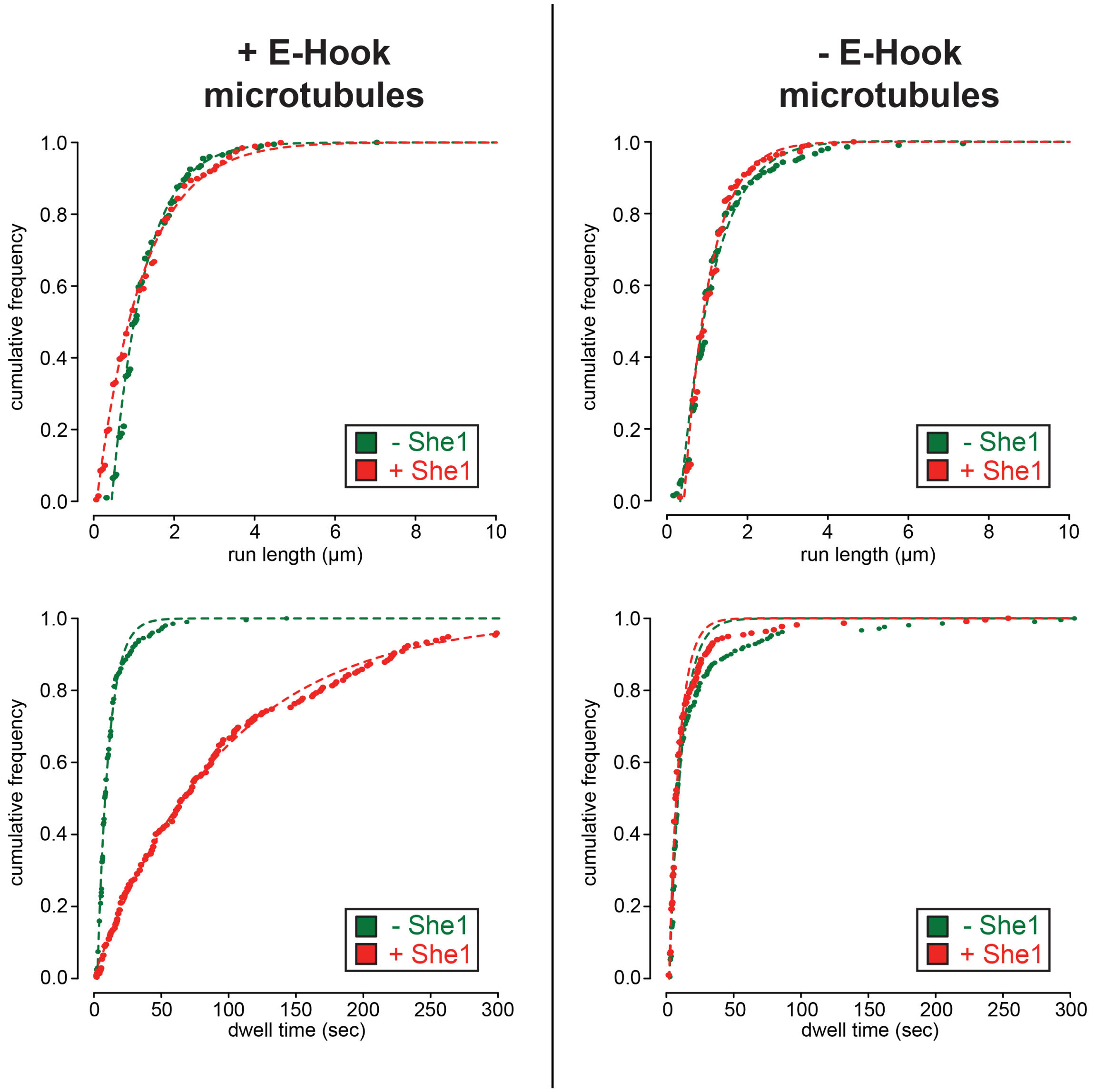

Supplementary Figure 2. **Cumulative probability functions used for determination of mean run length and dwell time values of dynein with and without She1 on control and subtilisin-treated microtubules.** Raw data (circles) and fits (dashed lines) are shown for run length (top) and dwell time (bottom) in the absence (green) and presence of She1 (red) on control (left) or subtilisin-treated microtubules (right; n ≥ 199 individual motors for each condition). Data were fit as previously described[2].

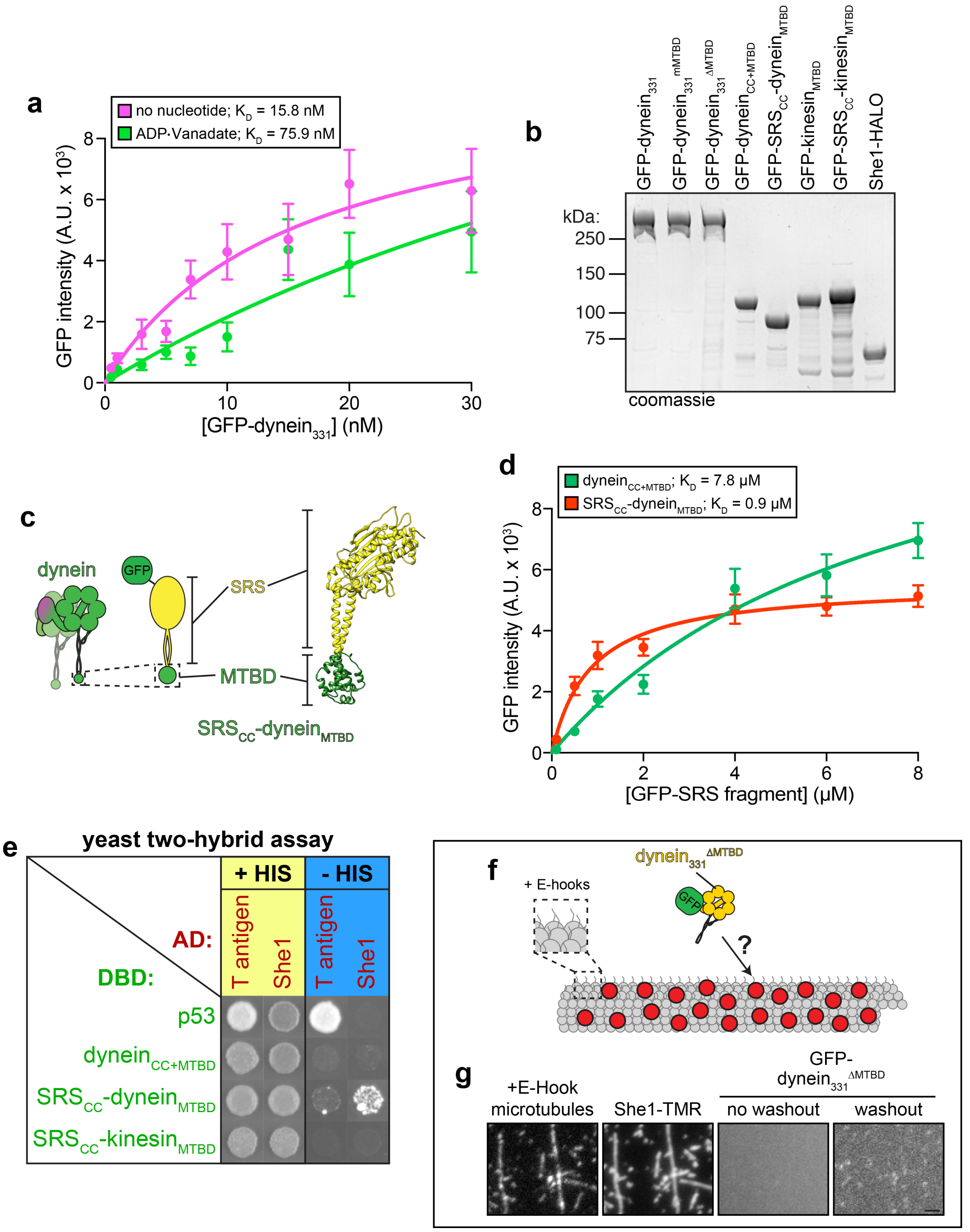

Supplementary Figure 3. **Characterization of recombinant fragments used in the recruitment assays, and two-hybrid data.** (a) Mean fluorescence intensity values (along with standard deviations) of microtubule-bound monomeric GFP-dynein$_{331}$ in the absence (magenta) or presence of ATP and vanadate (green). (b) Recombinant protein fragments used in the recruitment assays. With the exception of the dynein motor domain fragments, which were purified from yeast, all proteins were purified from *E. coli* (see Methods). (c) Cartoon representation

and reconstructed structural model of the SRS_{CC}-$dynein_{MTBD}$ fusion. Image was generated from a yeast model of the *DYN1* MTBD (threaded into 3ERR[3]) and 1SRY[4]. (d) Mean fluorescence intensity values (along with standard deviations) of microtubule-bound GFP-SRS_{CC}-$dynein_{MTBD}$ (red) and GFP-$dynein_{CC+MTBD}$ (green; n ≥ 19 microtubules, and ≥ 151 μm of MT length for each condition) along with fits and resulting dissociation constants (K_D). (e) Two-hybrid assay demonstrating an interaction between $dynein_{MTBD}$ and She1 (see Methods). (f) Schematic representation of the experimental setup used for panel g. (g) Representative images depicting the inability of microtubule-bound She1 to recruit GFP-$dynein_{331}{}^{\Delta MTBD}$ to microtubules. Images were acquired prior and subsequent to washing the chamber with motility buffer (see Methods; scale bar, 1 μm).

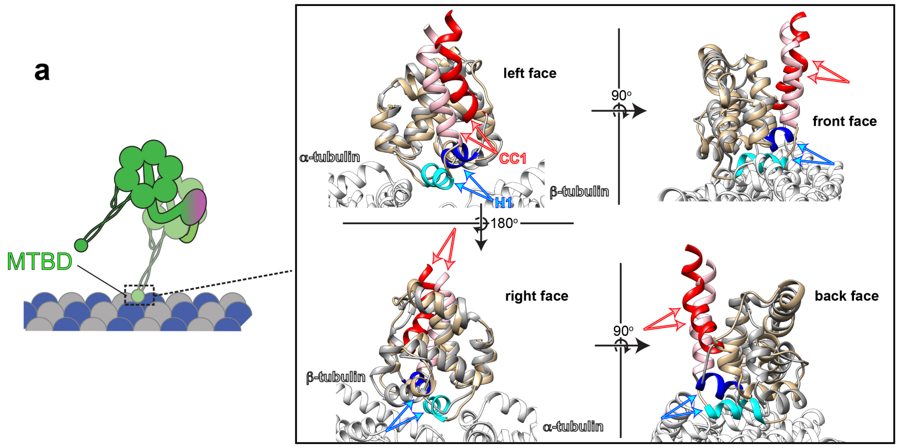

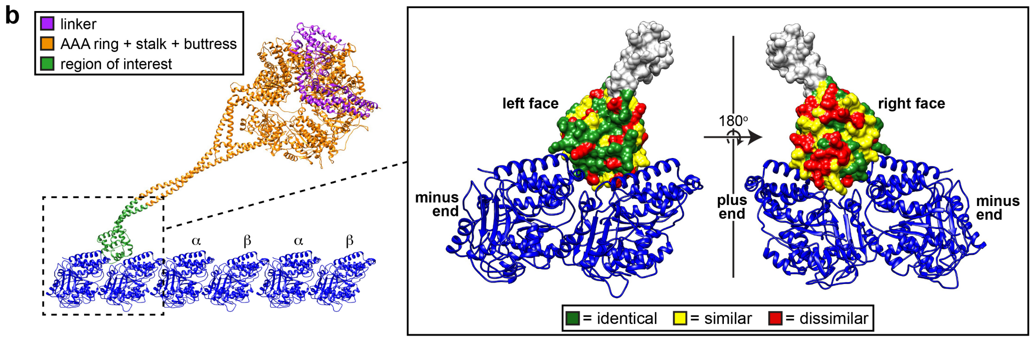

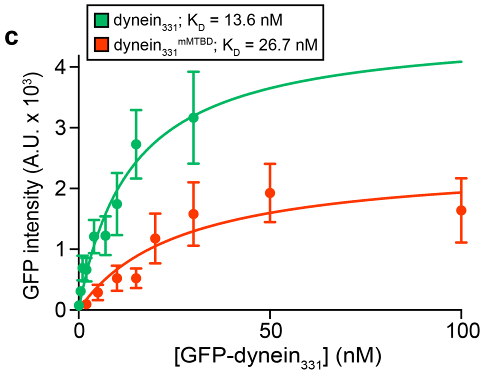

Supplementary Figure 4. **Comparison of various dynein MTBDs.** (a) Cartoon and homology models of the yeast dynein MTBD bound to α and β-tubulin in the high (grey) and low (tan) microtubule affinity states. The models were generated using one-to-one threading of the yeast *DYN1* sequence into 3J1T[5] (high affinity) and 3J1U (low affinity). CC1 and H1, which exhibit the largest differences between the two structures, are depicted as follows: CC1, red and pink, for high and low affinity states; H1, blue and cyan, for high and low affinity states, respectively. (b, left) Crystal structure of human dynein-2 (4RH7) docked onto microtubules (from 3J1T). (right) Homology model of the yeast MTBD (colored) – along with a short region of the CC (grey) – bound to α and β-tubulin in the high

microtubule affinity state. The residues are colored to reflect the degree of conservation between yeast and mouse primary sequence (see legend). (c) Mean fluorescence intensity values (along with standard deviations) of microtubule-bound GFP-dynein$_{331}$ (green) and GFP-dynein$_{331}^{mMTBD}$ (red; $n \geq 15$ microtubules, and ≥ 68 μm of MT length for each condition) along with fits and resulting dissociation constants (K_D). Note the differences in apparent B_{max} values (4645 ± 763 A.U. for wild-type, and 2452 ± 517 A.U. for mMTBD; ± SE of fit) are likely a consequence of microtubule unbinding during the chamber washes (see Methods), and likely differences in microtubule dissociation rates between the two motor domains.

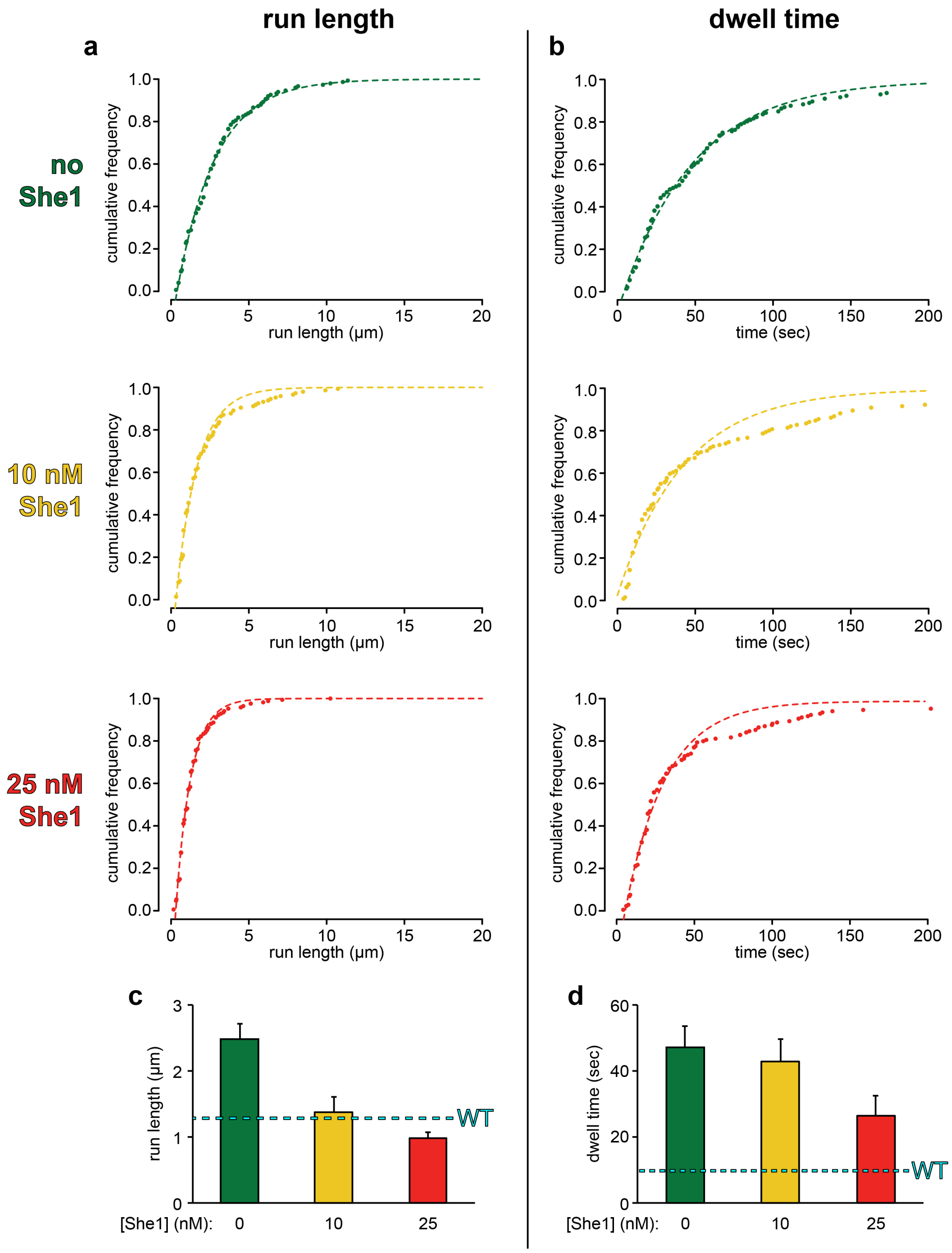

Supplementary Figure 5. **Cumulative probability functions and resulting mean values for run length and dwell time values of GST-dynein^{mMTBD} with and without She1.** (a and b) Raw data (circles) and fits (dashed lines) are shown for run length (a) and dwell time (b) in the absence (green) and presence of indicated concentrations of She1 (yellow and red; n ≥ 147 individual motors for each condition). Data were fit as previously described[2]. (c and d) Mean run length (c) and dwell time (d) for GST-dynein$_{331}^{mMTBD}$ in the presence of the indicated concentration of She1 (error bars, standard error). Dashed line indicates the mean run length or dwell time for wild-type (WT) GST-dynein$_{331}$ in the absence of She1.

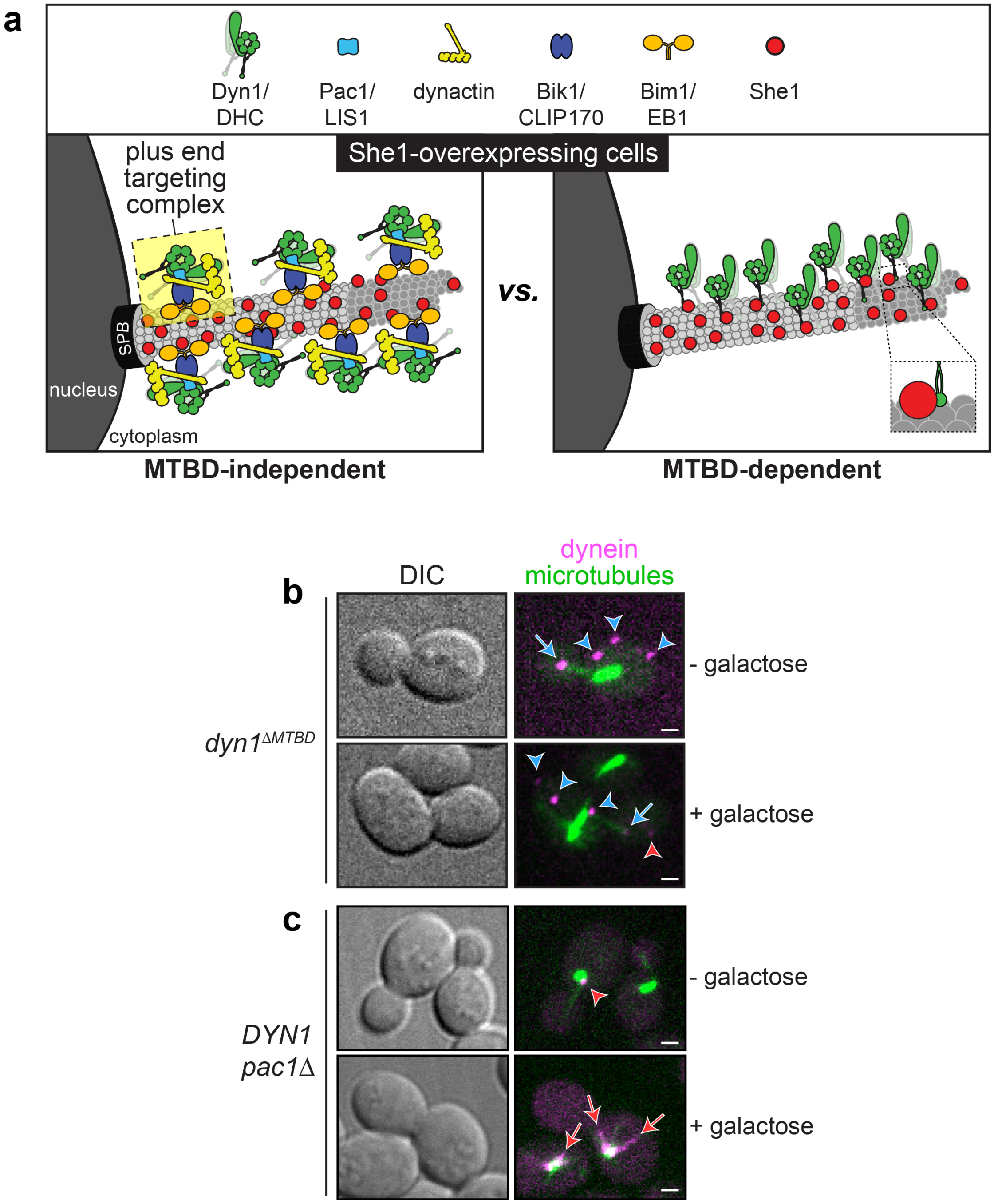

Supplementary Figure 6. **Dynein relocalization to astral microtubules upon She1 overexpression requires the dynein MTBD, but not Pac1.** (a) Cartoon representation of the two possible models to account for dynein relocalization upon She1 overexpression. The model on the left depicts a mechanism whereby the entire plus end targeting complex (composed of Dyn1, Pac1, Bik1 and Bim1; note that dynactin is not an obligate component of this complex[6]) is required for the relocalization. Given the dispensable nature for the MTBD in plus end targeting[7], this would indicate an MTBD-independent mechanism. The model on the right depicts a mechanism whereby dynein microtubule binding activity via

the MTBD is required. (b and c) Representative images of *GAL1p:SHE1* cells expressing mTurquoise2-Tub1 (b), or mRuby2-Tub1 (c), and either Dyn1$^{\Delta MTBD}$-3YFP (b) or Dyn1-3GFP (c), the latter of which is deleted for *PAC1*. Cells were grown to mid-log phase in SD media supplemented with raffinose (uninduced; "- galactose") or galactose plus raffinose (induced for 3.5 hours; "+ galactose") and then mounted on agarose pads for confocal fluorescence microscopy. Foci were identified in two-color movies and scored accordingly (see Methods; blue arrows, plus end foci; blue arrowheads, cortical foci; red arrowhead, dynamic cytoplasmic foci not associated with microtubules or spindle poles). Note the accumulation of Dyn1 near the spindle poles in *pac1Δ GAL1p:SHE1* cells grown in galactose-containing media (the same was observed in *GAL1p:SHE1 PAC1* cells; not shown). Movies reveal these spots exhibit dynamic movements in a manner that is consistent with them localizing to short astral microtubules, and not the spindle poles themselves. Note that our data support the MTBD-dependent model, depicted in panel a, right.

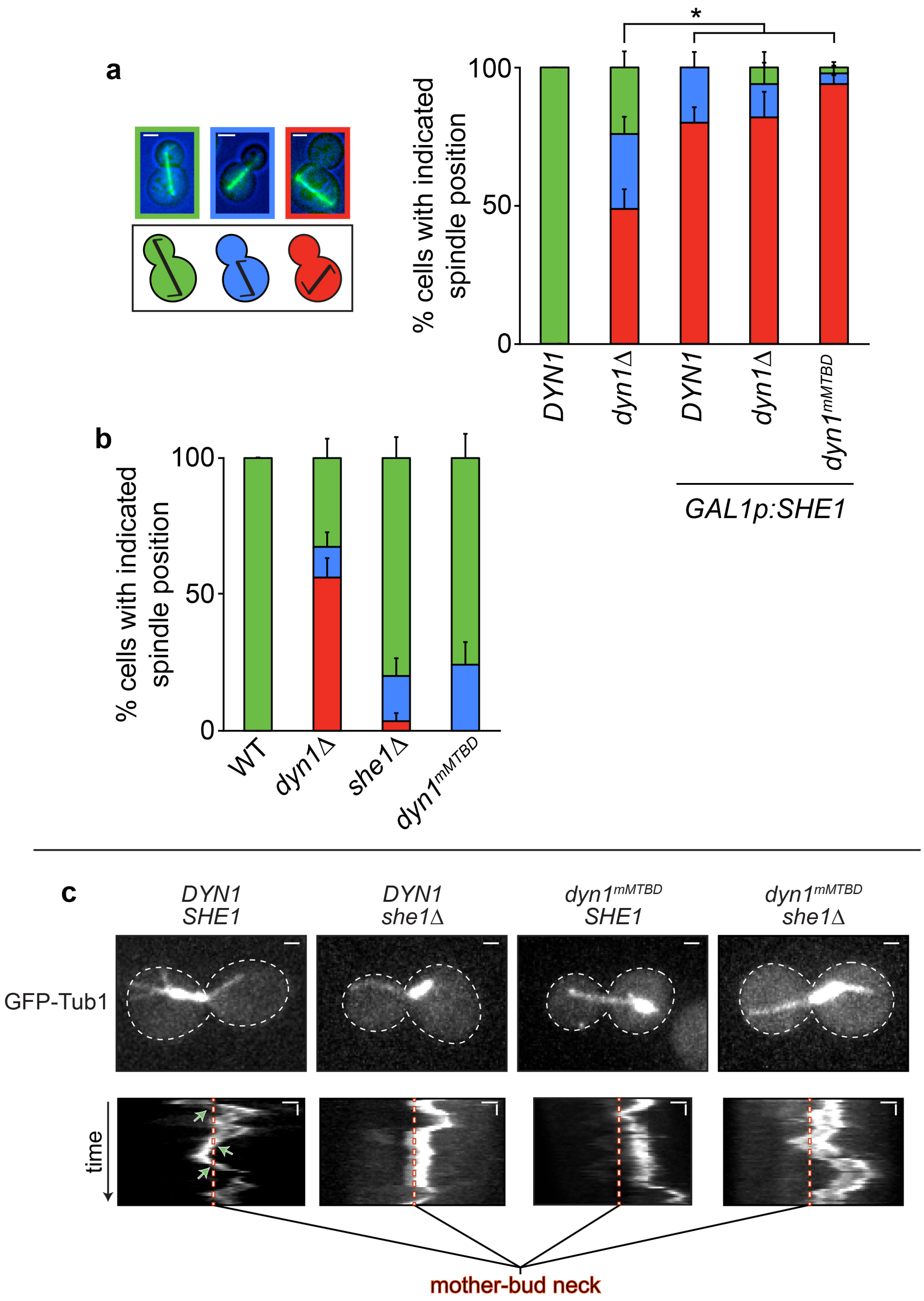

Supplementary Figure 7. ***In vivo* assessment of dynein^{mMTBD} mutant function.** (a and b) The percentage of cells with the indicated spindle orientation phenotype (green, normal; blue, aligned along mother-bud axis, but not through the neck; red, improperly aligned) is plotted for the indicated yeast strains (WT, wild-type). Anaphase spindles were visualized using mRuby2-Tub1 (α-tubulin). Strains were imaged after growth to mid-log phase in SD media supplemented with either (a) 2% galactose, or (b) 2% glucose, the former of which induces overexpression of She1 in *GAL1p:SHE1* cells (scale bars, 2 μm; error bars,

standard error of proportion; n ≥ 17 and n ≥ 21 anaphase spindles for each strain in panels a and b, respectively). Note the higher prevalence of misoriented spindles in She1-overexpressing cells (*GAL1p:SHE1*) than in cells lacking dynein (*dyn1*Δ; *, p ≤ 0.015). This suggests that She1-overexpression disrupts other non-dynein-mediated spindle orientation processes (*e.g.*, Kar9 pathway[8]). (c) Representative fluorescence images of *kar9*Δ, hydroxyurea (HU)-arrested GFP-Tub1 (α-tubulin) expressing cells with the indicated *SHE1* and *DYN1* alleles (scale bars, 1 μm), along with kymographs depicting spindle movements over time (horizontal scale bars, 1 μm; vertical scale bars, 1 min). Dashed lines indicate the position of the bud neck in each example. Note the frequency with which the spindle traverses the bud neck in wild-type, but not mutant cells (green arrows; see Fig. 7g for quantitation). P-values were calculated using a two-tailed unpaired *t* test.

Supplementary Table 1: Strains used in this study.

Strain	Genotype	Yeast background	Source
SMY193	Mata kar9Δ::KAN^R GFP-TUB1::LEU2 ura3-52 lys2-801 leu2-Δ1 his3-Δ200 trp1-Δ63	YEF473	This study
SMY263	Matα $dyn1^{rMTBD}$-3YFP::TRP1 TUB1+3'UTR::HPH::HIS3p:mRuby2-TUB1 ura3-52 lys2-801 leu2-Δ1 his3-Δ200 trp1-Δ63	YEF473	This study
SMY272	Matα $dyn1^{rMTBD}$-3YFP::TRP1 HIS3::GAL1p:SHE1 TUB1+3'UTR::HPH::HIS3p:mRuby2-TUB1 ura3-52 lys2-801 leu2-Δ1 his3-Δ200 trp1-Δ63	YEF473	This study
SMY293	Mata TUB1+3'UTR::HPH::HIS3p:mRuby2-TUB1 ura3-52 lys2-801 leu2-Δ1 his3-Δ200 trp1-Δ63	YEF473	This study
SMY295	Mata dyn1Δ::TRP1 TUB1+3'UTR::HPH::HIS3p:mRuby2-TUB1 ura3-52 lys2-801 leu2-Δ1 his3-Δ200 trp1-Δ63	YEF473	This study
SMY628	Matα $dyn1^{rMTBD}$-3YFP::TRP1 she1Δ::HIS3 kar9Δ::KAN^R GFP-TUB1::LEU2 ura3-52 lys2-801 leu2-Δ1 his3-Δ200 trp1-Δ63	YEF473	This study
SMY714	Matα $dyn1^{rMTBD}$-3YFP::TRP1 kar9Δ::KAN^R GFP-TUB1::LEU2 ura3-52 lys2-801 leu2-Δ1 his3-Δ200 trp1-Δ63	YEF473	This study
SMY950	Mata DYN1-3YFP::TRP1 JNM1-3mCherry::HIS3 she1Δ::URA3 TUB1+3'UTR::HPH::HIS3p:mRuby2-TUB1 ura3-52 lys2-801 leu2-Δ1 his3-Δ200 trp1-Δ63	YEF473	This study
SMY1006	Mata GAL1p:ZZ-TEV-6xHis-GFP-3XHA-fSNAPgs-$dyn1_{331}$-gsDHA::KAN^R prb1Δ pep4Δ3::HIS5 his3-11,15 ura3-52 leu2-3,112 ade2-1 trp-1	W303	This study
SMY1008	Mata GAL1p:ZZ-TEV-6xHis-GFP-3XHA-GST-$dyn1_{331}$-gsDHA::KAN^R prb1Δ pep4Δ3::HIS5 his3-11,15 ura3-52 leu2-3,112 ade2-1 trp-1	W303	This study
SMY1049	Mata GAL1p:ZZ-TEV-6xHis-GFP-3XHA-fSNAPgs-$dyn1_{331}^{\Delta MTBD}$-gsDHA::KAN^R prb1Δ pep4Δ3::HIS5 his3-11,15 ura3-52 leu2-3,112 ade2-1 trp-1	W303	This study
SMY1051	Mata GAL1p:ZZ-TEV-6xHis-GFP-3XHA-GST-$dyn1_{331}^{mMTBD}$-gsDHA::KAN^R prb1Δ pep4Δ3::HIS5 his3-11,15 ura3-52 leu2-3,112 ade2-1 trp-1	W303	This study
SMY1377	Mata GAL1p:ZZ-TEV-6xHis-GFP-3XHA-fSNAPgs-$dyn1_{331}^{mMTBD}$-gsDHA::KAN^R prb1Δ pep4Δ3::HIS5 his3-11,15 ura3-52 leu2-3,112 ade2-1 trp-1	W303	This study
SMY1427	Matα DYN1-3YFP::TRP KAN^R::GAL1p:SHE1 TUB1+3'UTR::HPH::HIS3p:mRuby2-TUB1 ura3-52 lys2-801 leu2-Δ1 his3-Δ200 trp1-Δ63	YEF473	This study
SMY1475	Matα $dyn1^{\Delta MTBD}$-3YFP::TRP1 KAN^R::GAL1p:SHE1 TUB1+3'UTR::HPH::HIS3p:mTurquoise2-TUB1 ura3-52 lys2-801 leu2-Δ1 his3-Δ200 trp1-Δ63	YEF473	This study
SMY1522	Mata DYN1-3GFP::TRP1 KAN^R::GAL1p:SHE1 pac1Δ:: HIS3 TUB1+3'UTR::HPH::HIS3p:mRuby2-TUB1 ura3-52 lys2-801 leu2-Δ1 his3-Δ200 trp1-Δ63	YEF473	This study
SMY1554	Matα kar9Δ::KAN^R she1Δ::HIS3 GFP-TUB1::LEU2 ura3-52 lys2-801 leu2-Δ1 his3-Δ200 trp1-Δ63	YEF473	This study

SMY1664	*Mata/α [pGBKT7-53]::TRP1 [pRS315:SHE1p:GAL4-AD-SHE1]::LEU2 LYS2::GAL1uas–Gal1tata:HIS3 GAL2uas–Gal2tata:ADE2/ade2-101 MEL1/MEL1 trp1-901/trp1-901 leu2-3,112/leu2-3,112 ura3-52::URA3::MEL1uas–Mel1tata:AUR1-C/ura3-52::URA3::GAL1uas–Gal1tata:LacZ his3-200/his3-200 gal4Δ/gal4Δ gal80Δ/gal80Δ met-*	Y2HGold/ Y187	This study
SMY1665	*Mata/α [pGBKT7:SRS-SRS$_{CC}$-dynein$_{MTBD}$]::TRP1 [pGADT7-T]::LEU2 LYS2::GAL1uas–Gal1tata:HIS3 GAL2uas–Gal2tata:ADE2/ade2-101 MEL1/MEL1 trp1-901/trp1-901 leu2-3,112/leu2-3,112 ura3-52::URA3::MEL1uas–Mel1tata:AUR1-C/ura3-52::URA3::GAL1uas–Gal1tata:LacZ his3-200/his3-200 gal4Δ/gal4Δ gal80Δ/gal80Δ met-*	Y2HGold/ Y187	This study
SMY1666	*Mata/α [pGBKT7:SRS-SRS$_{CC}$-dynein$_{MTBD}$]::TRP1 [pRS315:SHE1p:GAL4-AD-SHE1]::LEU2 LYS2::GAL1uas–Gal1tata:HIS3 GAL2uas–Gal2tata:ADE2/ade2-101 MEL1/MEL1 trp1-901/trp1-901 leu2-3,112/leu2-3,112 ura3-52::URA3::MEL1uas–Mel1tata:AUR1-C/ura3-52::URA3::GAL1uas–Gal1tata:LacZ his3-200/his3-200 gal4Δ/gal4Δ gal80Δ/gal80Δ met-*	Y2HGold/ Y187	This study
SMY1669	*Mata/α [pGBKT7:SRS-dynein$_{CC+MTBD}$]::TRP1 [pGADT7-T]::LEU2 LYS2::GAL1uas–Gal1tata:HIS3 GAL2uas–Gal2tata:ADE2/ade2-101 MEL1/MEL1 trp1-901/trp1-901 leu2-3,112/leu2-3,112 ura3-52::URA3::MEL1uas–Mel1tata:AUR1-C/ura3-52::URA3::GAL1uas–Gal1tata:LacZ his3-200/his3-200 gal4Δ/gal4Δ gal80Δ/gal80Δ met-*	Y2HGold/ Y187	This study
SMY1670	*Mata/α [pGBKT7:SRS-dynein$_{CC+MTBD}$]::TRP1 [pRS315:SHE1p:GAL4-AD-SHE1]::LEU2 LYS2::GAL1uas–Gal1tata:HIS3 GAL2uas–Gal2tata:ADE2/ade2-101 MEL1 MEL1 trp1-901/trp1-901 leu2-3,112/leu2-3,112 ura3-52::URA3::MEL1uas–Mel1tata:AUR1-C/ura3-52::URA3::GAL1uas–Gal1tata:LacZ his3-200/his3-200 gal4Δ/gal4Δ gal80Δ/gal80Δ met-*	Y2HGold/ Y187	This study
SMY1707	*Matα dyn1Δ::HIS3 KAN^R::GAL1p:SHE1 TUB1+3'UTR::LEU2::HIS3p:mRuby2-TUB1 ura3-52 lys2-801 leu2-Δ1 his3-Δ200 trp1-Δ63*	YEF473	This study
SMY1709	*Mata/α [pGBKT7-53]::TRP1 [pGADT7-T]::LEU2 LYS2::GAL1uas–Gal1tata:HIS3 GAL2uas–Gal2tata:ADE2/ade2-101 MEL1/MEL1 trp1-901/trp1-901 leu2-3,112/leu2-3,112 ura3-52::URA3::MEL1uas–Mel1tata:AUR1-C/ura3-52::URA3::GAL1uas–Gal1tata:LacZ his3-200/his3-200 gal4Δ/gal4Δ gal80Δ/gal80Δ met-*	Y2HGold/ Y187	This study

SMY1710	*Mata/α [pGBKT7:SRS-SRS*$_{CC}$*-dynein*$_{mMTBD}$*]::TRP1 [pGADT7-T]::LEU2 LYS2::GAL1uas–Gal1tata:HIS3 GAL2uas–Gal2tata:ADE2/ade2-101 MEL1/MEL1 trp1-901/trp1-901 leu2-3,112/leu2-3,112 ura3-52::URA3::MEL1uas–Mel1tata:AUR1-C/ura3-52::URA3::GAL1uas–Gal1tata:LacZ his3-200/his3-200 gal4Δ/gal4Δ gal80Δ/gal80Δ met-*	Y2HGold/ Y187	This study
SMY1711	*Mata/α [pGBKT7:SRS-SRS*$_{CC}$*-dynein*$_{mMTBD}$*]::TRP1 [pRS315:SHE1p:GAL4-AD-SHE1]::LEU2 LYS2::GAL1uas–Gal1tata:HIS3 GAL2uas–Gal2tata:ADE2/ade2-101 MEL1/MEL1 trp1-901/trp1-901 leu2-3,112/leu2-3,112 ura3-52::URA3::MEL1uas–Mel1tata:AUR1-C/ura3-52::URA3::GAL1uas–Gal1tata:LacZ his3-200/his3-200 gal4Δ/gal4Δ gal80Δ/gal80Δ met-*	Y2HGold/ Y187	This study
SMY1712	*Mata/α [pGBKT7:SRS-SRS*$_{CC}$*-kinesin*$_{MTBD}$*]::TRP1 [pGADT7-T]::LEU2 LYS2::GAL1uas–Gal1tata:HIS3 GAL2uas–Gal2tata:ADE2/ade2-101 MEL1/MEL1 trp1-901/trp1-901 leu2-3,112/leu2-3,112 ura3-52::URA3::MEL1uas–Mel1tata:AUR1-C/ura3-52::URA3::GAL1uas–Gal1tata:LacZ his3-200/his3-200 gal4Δ/gal4Δ gal80Δ/gal80Δ met-*	Y2HGold/ Y187	This study
SMY1713	*Mata/α [pGBKT7:SRS-SRS*$_{CC}$*-kinesin*$_{MTBD}$*]::TRP1 [pRS315:SHE1p:GAL4-AD-SHE1]::LEU2 LYS2::GAL1uas–Gal1tata:HIS3 GAL2uas–Gal2tata:ADE2/ade2-101 MEL1/MEL1 trp1-901/trp1-901 leu2-3,112/leu2-3,112 ura3-52::URA3::MEL1uas–Mel1tata:AUR1-C/ura3-52::URA3::GAL1uas–Gal1tata:LacZ his3-200/his3-200 gal4Δ/gal4Δ gal80Δ/gal80Δ met-*	Y2HGold/ Y187	This study

*rMTBD (MTBD obtained from rat DHC) is identical to mMTBD in amino acid sequence

Supplementary References

1. Yildiz, A., Forkey, J. N., McKinney, S. A., Ha, T., Goldman, Y. E. & Selvin, P. R. Myosin V walks hand-over-hand: single fluorophore imaging with 1.5-nm localization. Science. 2003;300(5628):2061-2065.
2. Reck-Peterson, S. L., Yildiz, A., Carter, A. P., Gennerich, A., Zhang, N. & Vale, R. D. Single-molecule analysis of dynein processivity and stepping behavior. Cell. 2006;126(2):335-348.
3. Carter, A. P., Garbarino, J. E., Wilson-Kubalek, E. M., Shipley, W. E., Cho, C., Milligan, R. A., Vale, R. D. & Gibbons, I. R. Structure and functional role of dynein's microtubule-binding domain. Science. 2008;322(5908):1691-1695.
4. Fujinaga, M., Berthet-Colominas, C., Yaremchuk, A. D., Tukalo, M. A. & Cusack, S. Refined crystal structure of the seryl-tRNA synthetase from Thermus thermophilus at 2.5 A resolution. J Mol Biol. 1993;234(1):222-233.
5. Redwine, W. B., Hernandez-Lopez, R., Zou, S., Huang, J., Reck-Peterson, S. L. & Leschziner, A. E. Structural basis for microtubule binding and release by dynein. Science. 2012;337(6101):1532-1536.
6. Lee, W. L., Oberle, J. R. & Cooper, J. A. The role of the lissencephaly protein Pac1 during nuclear migration in budding yeast. J Cell Biol. 2003;160(3):355-364.
7. Lammers, L. G. & Markus, S. M. The dynein cortical anchor Num1 activates dynein motility by relieving Pac1/LIS1-mediated inhibition. J Cell Biol. 2015;211(2):309-322.
8. Markus, S. M., Kalutkiewicz, K. A. & Lee, W. L. Astral microtubule asymmetry provides directional cues for spindle positioning in budding yeast. Experimental cell research. 2012;318(12):1400-1406.
